# Supplementary material for: Evidence from stable isotopes and 10Be for solar system formation triggered by a low-mass supernova
Source: Nat Commun. 2016 Nov 22;7:13639. doi: 10.1038/ncomms13639 (PMC5121422; doi:10.1038/ncomms13639)
Supplement: Supplementary Information — Supplementary Figure 1, Supplementary Tables 1-4, Supplementary Discussion and Supplementary References. [file ncomms13639-s1.pdf]

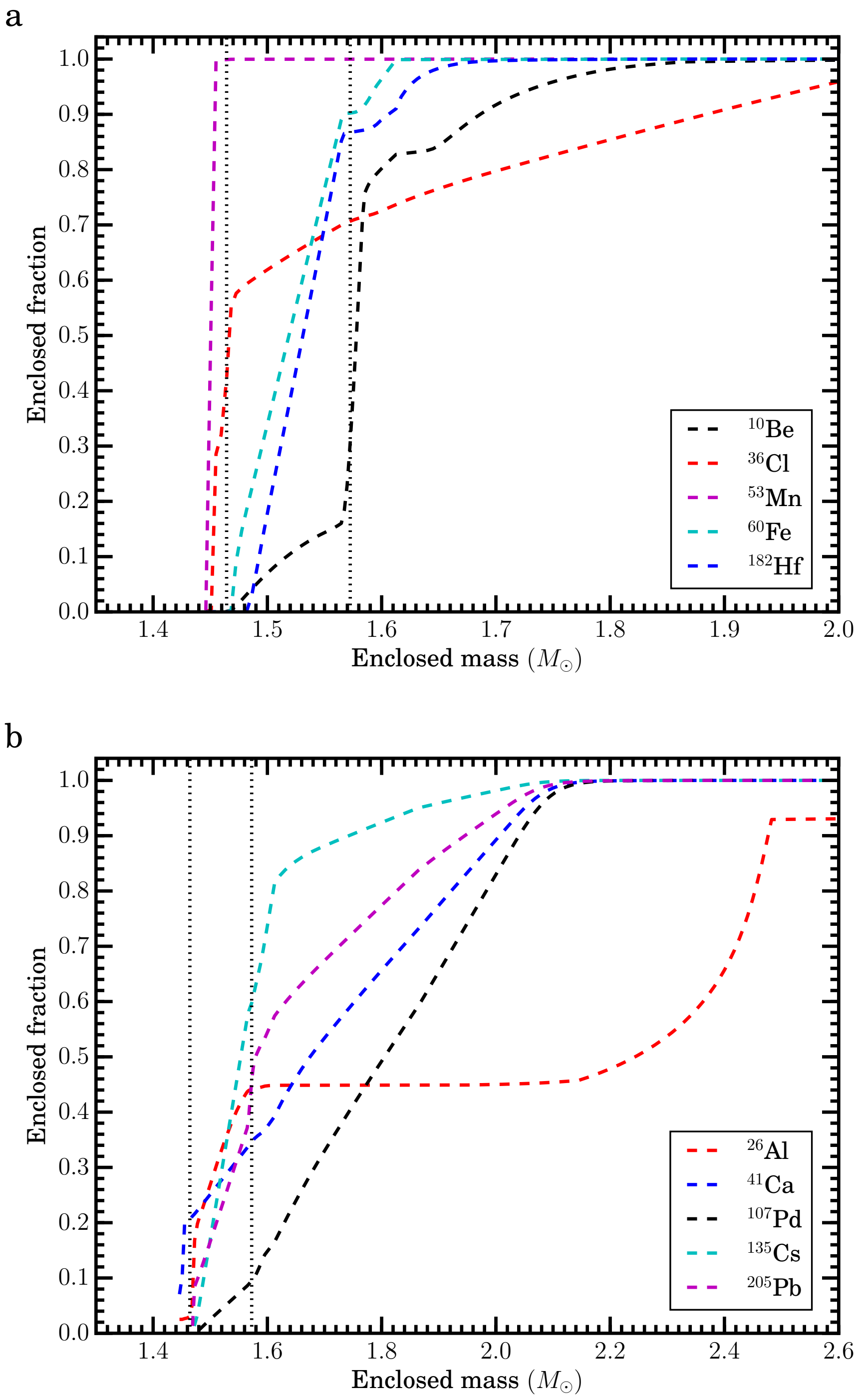

Supplementary Figure 1: Enclosed fraction of the total yield of each short-lived radionuclide as a function of the enclosed stellar mass for the 11.8-solar-mass model with no fallback (Case 1). The left and right vertical lines indicate the boundary between fallback and ejected material for Cases 2 and 3, respectively.

Supplementary Table 1: Supernova contributions to short-lived radionuclides in the early solar system from the 20- and 30-solar-mass models

R/I	$20\,M_\odot$	$30\,M_\odot$
^{10}Be/^{9}Be	7.39(−5)	3.70(−5)
^{26}Al/^{27}Al	5.64(−5)	5.17(−5)
^{36}Cl/^{35}Cl	2.89(−6)	1.37(−6)
^{41}Ca/^{40}Ca	4.95(−9)	4.21(−9)
^{53}Mn/^{55}Mn	6.24(−6)	6.40(−6)
^{60}Fe/^{56}Fe	1.04(−6)	9.48(−7)
^{107}Pd/^{108}Pd	3.02(−4)	1.47(−4)
^{135}Cs/^{133}Cs	3.01(−4)	5.85(−5)
^{182}Hf/^{180}Hf	4.54(−5)	7.52(−6)
^{205}Pb/^{204}Pb	4.76(−04)	1.35(−4)

Fallback scenarios similar to those of Ref. [1] are assumed. Values for ^{182}Hf are obtained from the estimated stellar decay rate of ^{181}Hf [2]. Note that ^{107}Pd is overproduced and $x(-y)$ denotes $x \times 10^{-y}$.

Supplementary Table 2: Yields of major stable isotopes for the 11.8-solar-mass model with no fallback (Case 1)

Isotope	Yield ($M_\odot$)	Isotope	Yield ($M_\odot$)	Isotope	Yield ($M_\odot$)
^{12}C	5.75(−2)	^{33}S	3.39(−5)	^{54}Cr	5.08(−6)
^{13}C	6.98(−4)	^{34}S	1.91(−4)	^{54}Fe	8.58(−4)
^{14}N	2.68(−2)	^{36}S	1.18(−6)	^{56}Fe	1.77(−2)
^{15}N	1.96(−5)	^{40}Ca	7.28(−4)	^{57}Fe	5.51(−4)
^{16}O	1.43(−1)	^{42}Ca	4.00(−6)	^{58}Fe	9.23(−5)
^{17}O	5.41(−5)	^{43}Ca	1.05(−6)	^{58}Ni	6.95(−4)
^{18}O	7.46(−4)	^{44}Ca	1.64(−5)	^{60}Ni	3.53(−4)
^{20}Ne	3.55(−2)	^{46}Ca	5.36(−8)	^{61}Ni	2.76(−5)
^{21}Ne	1.17(−4)	^{48}Ca	1.21(−6)	^{62}Ni	8.73(−5)
^{22}Ne	3.16(−3)	^{46}Ti	2.22(−6)	^{64}Ni	2.00(−5)
^{24}Mg	8.60(−3)	^{47}Ti	2.29(−6)	^{64}Zn	1.03(−5)
^{25}Mg	1.50(−3)	^{48}Ti	3.03(−5)	^{66}Zn	8.75(−6)
^{26}Mg	1.61(−3)	^{49}Ti	2.19(−6)	^{67}Zn	1.40(−6)
^{28}Si	9.11(−3)	^{50}Ti	1.80(−6)	^{68}Zn	5.64(−6)
^{29}Si	4.38(−4)	^{50}Cr	9.32(−6)	^{70}Zn	1.63(−7)
^{30}Si	4.29(−4)	^{52}Cr	2.15(−4)		
^{32}S	4.71(−3)	^{53}Cr	2.74(−5)		

Note that $x(-y)$ denotes $x \times 10^{-y}$.

Supplementary Table 3: Percentage of the solar system inventory of major stable isotopes contributed by the 11.8-solar-mass model

Isotope	Case 1	Case 2	Case 3	Isotope	Case 1	Case 2	Case 3
^{12}C	1.16	1.16	1.10	^{48}Ca	0.45	0.45	0.44
^{13}C	1.16	1.16	1.16	^{46}Ti	0.46	0.46	0.45
^{14}N	1.82	1.82	1.82	^{47}Ti	0.52	0.45	0.44
^{15}N	0.33	0.33	0.22	^{48}Ti	0.68	0.44	0.44
^{16}O	1.18	1.18	0.56	^{49}Ti	0.65	0.51	0.48
^{17}O	1.10	1.10	1.06	^{50}Ti	0.55	0.55	0.48
^{18}O	2.72	2.72	2.72	^{50}Cr	0.63	0.44	0.44
^{20}Ne	1.44	1.44	0.48	^{52}Cr	0.73	0.45	0.44
^{21}Ne	1.89	1.89	0.98	^{53}Cr	0.80	0.45	0.45
^{22}Ne	1.59	1.59	1.56	^{54}Cr	0.59	0.59	0.50
^{24}Mg	0.86	0.86	0.45	^{54}Fe	0.62	0.44	0.44
^{25}Mg	1.14	1.14	0.51	^{56}Fe	0.79	0.45	0.44
^{26}Mg	1.07	1.07	0.66	^{57}Fe	1.04	0.50	0.48
^{28}Si	0.70	0.57	0.44	^{58}Fe	1.29	1.29	0.73
^{29}Si	0.64	0.64	0.46	^{58}Ni	0.71	0.44	0.44
^{30}Si	0.92	0.92	0.47	^{60}Ni	0.91	0.50	0.46
^{32}S	0.71	0.47	0.44	^{61}Ni	1.61	1.07	0.63
^{33}S	0.62	0.56	0.47	^{62}Ni	1.57	0.76	0.54
^{34}S	0.60	0.59	0.45	^{64}Ni	1.37	1.37	0.91
^{36}S	0.75	0.75	0.55	^{64}Zn	0.50	0.45	0.44
^{40}Ca	0.62	0.44	0.44	^{66}Zn	0.72	0.64	0.52
^{42}Ca	0.49	0.49	0.46	^{67}Zn	0.77	0.76	0.56
^{43}Ca	0.60	0.52	0.46	^{68}Zn	0.67	0.67	0.54
^{44}Ca	0.59	0.46	0.45	^{70}Zn	0.57	0.57	0.45
^{46}Ca	0.96	0.96	0.52				

Supplementary Table 4: Core-collapse supernova yields of stable Li, Be, and B isotopes for the 11.8-solar-mass model with no fallback (Case 1)

Isotope	Yield ($M_\odot$)	Case 1	Case 2	Case 3
^{6}Li	1.10(−11)	8.5(−4)	8.5(−4)	8.4(−4)
^{7}Li	1.55(−7)	0.84	0.83	0.82
^{9}Be	5.99(−11)	2.1(−2)	2.1(−2)	2.1(−2)
^{10}B	1.45(−9)	7.6(−2)	7.6(−2)	6.8(−2)
^{11}B	4.25(−7)	5.03	5.00	4.56

Corresponding percentages of the solar system inventories are given for Case 1 and two other cases with fallback. Note that $x(-y)$ denotes $x \times 10^{-y}$.

Supplementary Discussion

Core-collapse supernova (CCSN) yields of stable isotopes. Supplementary Table 2 gives the yields of major stable isotopes for the $11.8\,M_\odot$ CCSN model assuming no fallback (Case 1). As shown in Fig. 1a of the main text, the yields of stable isotopes increase greatly for CCSNe of 14–30 $M_\odot$. For a stable isotope ^{i}E of element E, the percentage of its solar system (SS) inventory contributed by a CCSN is

$$\eta({}^i\mathrm{E}) \sim \frac{fY({}^i\mathrm{E})}{X_\odot({}^i\mathrm{E})M_\odot} \times 100, \tag{1}$$

where $Y({}^i\mathrm{E})$ is its yield, f is the fraction incorporated into each $M_\odot$ of the protosolar cloud, and $X_\odot({}^i\mathrm{E})$ is its solar mass fraction. The CCSN contributions would introduce shifts in ${}^i\mathrm{E}/{}^j\mathrm{E}$, the number ratio of isotopes ^{i}E and ^{j}E, for SS materials. The percentage shift for macroscopic samples can be estimated as $\delta({}^i\mathrm{E}/{}^j\mathrm{E}) = 100[({}^i\mathrm{E}/{}^j\mathrm{E})/({}^i\mathrm{E}/{}^j\mathrm{E})_\odot - 1] \sim \eta({}^i\mathrm{E}) - \eta({}^j\mathrm{E})$, where $({}^i\mathrm{E}/{}^j\mathrm{E})_\odot$ is the SS average. No large shifts at the few percent level have been observed for stable isotopes of e.g., Mg, Si, Ca, Fe, and Ni, and the observed large excess in ^{16}O is most likely unrelated to its nucleosynthetic origin [3].

Supplementary Table 3 gives $\eta({}^i\mathrm{E})$ for Case 1 of the $11.8\,M_\odot$ model without fallback and Cases 2 and 3 with fallback, assuming $f \sim 5 \times 10^{-4}$ for all cases. It can be seen that in all cases the above CCSN contributed $\lesssim 1\%$ of the SS inventory for most of the major stable isotopes, with the highest contribution being 2.72% for ^{18}O. The shifts in ${}^i\mathrm{E}/{}^j\mathrm{E}$ due to this CCSN are entirely consistent with meteoritic constraints [3].

In Cases 2 and 3 of the $11.8\,M_\odot$ model with fallback, the percentage contributions to the heaviest Cr (^{54}Cr), Fe (^{58}Fe), and Ni (^{64}Ni) isotopes are the highest among their respective isotopes (see Supplementary Table 3). This arises because fallback is efficient in destroying the products of explosive nucleosynthesis, but has a smaller effect on the neutron-rich isotopes produced by the slow neutron-capture (s) process during pre-CCSN evolution. Macroscopic samples receiving contributions from the $11.8\,M_\odot$ CCSN would typically show excesses of ${}^{54}\mathrm{Cr}/{}^{52}\mathrm{Cr}$, ${}^{58}\mathrm{Fe}/{}^{56}\mathrm{Fe}$, and ${}^{64}\mathrm{Ni}/{}^{58}\mathrm{Ni}$ at levels of $\sim 10^{-3}$, similar to those observed in meteorites. As there are few satisfactory explanations of these excesses [4], this provides additional circumstantial support for the thesis that a low-mass CCSN with modest fallback triggered SS formation. We note that Ref. [4] explored a different explanation of these excesses by s-processing in asymptotic-giant-branch (AGB) stars. A

crucial distinction between the above two explanations lie in their associated grains that would carry much larger anomalies. Grains from a low-mass CCSN would show excesses of ^{29}Si/^{28}Si and ^{30}Si/^{28}Si (see especially Case 2 in Supplementary Table 3), but those from AGB stars would not [4].

CCSN production of short-lived radionuclides (SLRs). Most of the ^{10}Be production occurs in the C and O shells, where the mass fraction of ^{12}C is high but that of ^{4}He is low. A low ^{4}He abundance is crucial in avoiding ^{10}Be destruction via ^{10}Be$(\alpha, n)^{13}$C. For the same reason, neutrino-induced production of ^{10}Be is self-limiting because spallation of ^{12}C and ^{16}O also produces ^{4}He and protons that can destroy ^{10}Be via ^{10}Be$(p, \alpha)^{7}$Li. As long as abundances of ^{4}He and protons are low, most of the ^{10}Be survives even when the production zone is heated by shock passage. We note that Ref. [5] adopted a rate for ^{10}Be$(\alpha, n)^{13}$C that is orders of magnitude larger than currently recommended [6], and therefore, greatly underestimated the ^{10}Be yield. In addition, the $16.2\,M_\odot$ model used in that work was evolved from a helium core with a fitted hydrogen envelope [7] while each of our models has been evolved self-consistently as a whole star. We find that the radii of the C/O shell in that $16.2\,M_\odot$ model are ~ 2 times larger than those in our $16\,M_\odot$ model. Consequently, the neutrino flux for ^{10}Be production was significantly smaller in Ref. [5], which also contributed to the much smaller yield reported there.

The SLR ^{36}Cl can be produced by neutrinos via ^{36}Ar$(\bar{\nu}_e, e^+)^{36}$Cl, where the ^{36}Ar is made during shock passage by explosive nucleosynthesis. This channel accounts for $\sim 60\%$ of the ^{36}Cl yield in our $11.8\,M_\odot$ model. A significant fraction (up to $\sim 40\%$) of ^{26}Al is made by explosive nucleosynthesis via ^{25}Mg$(p, \gamma)^{26}$Al, including some enhancement by the protons released from neutrino spallation. ^{53}Mn is almost entirely a product of explosive nucleosynthesis in all of our models.

SLRs are also produced during pre-CCSN evolution: ^{26}Al through hydrostatic burning at the edge of the He shell; ^{36}Cl, ^{41}Ca, ^{107}Pd, and ^{205}Pb mainly through the s process associated with He core burning; and ^{60}Fe, ^{135}Cs, and ^{182}Hf mostly through the s process associated with C burning in the O shell. The neutrons for the s process come from ^{22}Ne$(\alpha, n)^{25}$Mg, while the seeds come from the progenitor's initial composition, which is taken as solar in all of our models.

For the $11.8\,M_\odot$ model with no fallback (Case 1), Supplementary Fig. 1 shows the fraction

of the total yield of each SLR enclosed within a specific radius, as a function of the stellar mass enclosed within the same radius. The left (right) vertical line indicates the boundary between fallback and ejected material for Case 2 (3) with only 1.5% of the innermost $1.02 \times 10^{-2}\,M_\odot$ ($0.116\,M_\odot$) of shocked material ejected. As the enclosed fraction of the ^{10}Be yield sharply increases near the boundary for Case 3, this case represents the limit of reducing ^{53}Mn and ^{60}Fe without affecting the concordance among ^{10}Be, ^{41}Ca, and ^{107}Pd shown in Fig. 2 of the main text.

Reference [1] used high-mass CCSN models with fallback to explain the meteoritic data on the SLRs ^{26}Al, ^{41}Ca, ^{53}Mn, and ^{60}Fe. Supplementary Table 1 gives the results for our $20\,M_\odot$ and $30\,M_\odot$ models based on similar scenarios. For the $20\,M_\odot$ ($30\,M_\odot$) model, we have used $f \sim 1.1 \times 10^{-3}$ (6.7×10^{-4}) and $\Delta \sim 1.2$ Myr (1.1 Myr) in equation (1) of the main text and assumed that a fraction $q \sim 5 \times 10^{-4}$ (8×10^{-4}) of the shocked material below the fallback boundary at the mass cut $M_{\rm cut} \sim 4.68\,M_\odot$ ($9.38\,M_\odot$) is ejected. The corresponding parameters in Ref. [1] are $f \sim 1.9 \times 10^{-3}$ (4.35×10^{-4}), $\Delta \sim 1.07$ Myr (0.87 Myr), $q \sim 10^{-3}$ (10^{-3}), and $M_{\rm cut} \sim 3.1\,M_\odot$ ($6.7\,M_\odot$). For our $20\,M_\odot$ ($30\,M_\odot$) model, contributions to the SS inventory of ^{16}O, ^{17}O, and ^{18}O are $\sim 3.1\%$, 1.7%, and 21.6% (0.3%, 0.4%, and 18.1%), respectively. These would have caused large shifts in ^{18}O/^{16}O and ^{17}O/^{18}O that are not observed [8]. Further, both our models overproduce ^{107}Pd (see Supplementary Table 1 and Table 1 of the main text). No calculation was presented for this SLR in Ref. [1].

With similar yields of ^{10}Be for CCSNe of 11.8–30 $M_\odot$, production by these sources against decay may maintain an inventory of ^{10}Be in the interstellar medium (ISM). An upper limit on the corresponding mass fraction can be estimated as

$$X_{\rm ISM}(^{10}{\rm Be}) \sim \frac{\langle Y(^{10}{\rm Be})\rangle R_{\rm SN}}{M_{\rm ISM}} \tau(^{10}{\rm Be}) \sim 10^{-14}, \tag{2}$$

where $\langle Y(^{10}{\rm Be})\rangle \sim 5 \times 10^{-10}\,M_\odot$ is the average yield (see Table 1 and Fig. 1b of the main text), $\tau(^{10}{\rm Be}) \sim 2$ Myr is the lifetime, and $R_{\rm SN} \sim (10\ {\rm yr})^{-1}$, the rate of CCSNe in a total mass $M_{\rm ISM} \sim 10^{10}\,M_\odot$ of ISM, is taken near the upper bound of plausible rates. The above estimate can be combined with the mass fraction of ^{9}Be in the ISM, $X_\odot(^9{\rm Be}) \sim 1.4 \times 10^{-10}$, to give $(^{10}{\rm Be}/^9{\rm Be})_{\rm ISM} \sim 6 \times 10^{-5}$ at the time of SS formation. This is ~ 10 times less than the typical value in the early SS (see Table 1 of the main text). As we have shown, the latter can be explained by a low-mass CCSN similar to our $11.8\,M_\odot$ model that triggered the formation of the SS.

<u>CCSN remnant evolution and SLRs in the early SS</u>. The triggering of SS formation and the injection of SLRs into the early SS by a low-mass CCSN depend on the evolution of its remnant. This evolution most likely occurred in a giant molecular cloud with gas of varying density surrounding much denser clumps. The protosolar cloud resided in the core of one of these clumps. For triggering the collapse of the protosolar cloud, the shock velocity associated with remnant expansion should have been $v_{\rm s} \sim 20$–40 km s^{-1} [9–11]. There is also a requirement on the remnant size so that a fraction $f \sim 5 \times 10^{-4}$ of the CCSN ejecta was incorporated into each $M_\odot$ of the protosolar cloud to account for the SLRs ^{10}Be, ^{41}Ca, and ^{107}Pd simultaneously. For a remnant with a shock radius $R_{\rm s}$ colliding with a cloud of radius $r_{\rm c}$, only a fraction $\sim r_{\rm c}^2/(4R_{\rm s}^2)$ of the remnant material would be available for injection. So f can be estimated as

$$f \sim \epsilon_{\rm in}\left(\frac{r_{\rm c}^2}{4R_{\rm s}^2}\right) \sim 2.5\times 10^{-4}\left(\frac{\epsilon_{\rm in}}{0.1}\right)\left(\frac{r_{\rm c}}{0.1\ {\rm pc}}\right)^2\left(\frac{\rm pc}{R_{\rm s}}\right)^2, \tag{3}$$

where $\epsilon_{\rm in}$ is the efficiency of injecting the relevant part of the remnant material into each $M_\odot$ of the protosolar cloud. Guided by simulations [9–11], we take $\epsilon_{\rm in} \sim 0.1$ for a typical clump core of $\sim 1\,M_\odot$ with $r_{\rm c} \sim 0.1$ pc [12]. Based on the above discussion, our proposed trigger scenario requires that the low-mass CCSN remnant must have had $v_{\rm s} \sim 20$–40 km s^{-1} and $R_{\rm s} \sim 1$ pc when colliding with the protosolar cloud. In addition, these conditions must have been reached within $\Delta \sim 1$ Myr to account for the pertinent SLRs, especially ^{41}Ca with a very short lifetime of ~ 0.15 Myr.

The shock velocities of concern typically occur when the remnant is in the pressure-driven snowplow (PDS) phase. For reference, we consider the simple case of a CCSN remnant expanding in a uniform ISM. At the onset of the PDS phase, the shock radius $R_{\rm PDS}$ and velocity $v_{\rm PDS}$ [13] are approximately given by

$$R_{\rm PDS} \sim 1.01\left(\frac{E}{10^{50}\ {\rm erg}}\right)^{2/7}\left(\frac{100\ {\rm cm}^{-3}}{n_0}\right)^{3/7}\ {\rm pc}, \tag{4}$$

$$v_{\rm PDS} \sim 676\left(\frac{E}{10^{50}\ {\rm erg}}\right)^{1/14}\left(\frac{n_0}{100\ {\rm cm}^{-3}}\right)^{1/7}\ {\rm km\ s}^{-1}, \tag{5}$$

where E is the explosion energy of the CCSN, and n_0 is the number density of hydrogen atoms in the ISM. The remnant evolution during the PDS phase [13] is approximately

described by

$$R_s \sim R_{PDS}\left(\frac{4}{3}t_* - \frac{1}{3}\right)^{3/10}, \tag{6}$$

$$v_s \sim v_{PDS}\left(\frac{4}{3}t_* - \frac{1}{3}\right)^{-7/10}, \tag{7}$$

where $t_* \equiv t/t_{PDS}$ is the time t since the explosion in units of

$$t_{PDS} \sim 584\left(\frac{E}{10^{50}\ \text{erg}}\right)^{3/14}\left(\frac{100\ \text{cm}^{-3}}{n_0}\right)^{4/7}\ \text{yr}. \tag{8}$$

Using $E \sim 10^{50}$ erg for the low-mass CCSN and $n_0 \sim 100$ cm^{-3} for a typical giant molecular cloud [12], we find that for the above simple case, the remnant reaches $v_s \sim 40$ km s^{-1} at $t \sim 2.5 \times 10^4$ yr. The corresponding $R_s \sim 3.4$ pc gives $f \sim 2 \times 10^{-5}$. This should be regarded as a lower limit on f because the shock wave can be slowed down more efficiently in a giant molecular cloud with dense clumps . For example, if at the onset of the PDS phase the remnant in the simple case encounters a clump with a hydrogen density of $n_0' \sim 2 \times 10^3$ cm^{-3} and a radius of ~ 1 pc [12], then by momentum conservation relevant for the PDS phase, the shock wave approaches the core of the clump with $v_s \sim (n_0/n_0')v_{PDS} \sim 34$ km s^{-1} but its effective radius remains close to $R_s \sim 1$ pc. In this example, the conditions for triggering the collapse of the core and injecting SLRs into it would be satisfied. Based on the above discussion, we consider our trigger scenario reasonable and urge that simulations of remnant evolution in a giant molecular cloud be carried out to provide more rigorous results. We note that the time of remnant expansion must have been far shorter than $\Delta \sim 1$ Myr. Therefore, this interval must reflect the timescales associated with the collapse of the protosolar cloud and the formation of the first solids in the early SS.

Evolution of a CCSN remnant prior to the PDS phase is associated with acceleration of cosmic rays (CRs), which can produce ^{10}Be [14]. We consider the amount of ^{10}Be produced by CRs inside the remnant up to the onset of the PDS phase and compare this to the low-mass CCSN yield. Using $n_0 \sim 100$ cm^{-3} but an explosion energy 10 times too high for the low-mass CCSN, Ref. [14] found that CRs can produce ^{10}Be/^{9}Be $\sim 2.5 \times 10^{-3}$ at the maximum. Adopting this upper limit and a total mass of swept-up ISM

$$M_{PDS} \sim 10\, M_\odot \left(\frac{n_0}{100\ \text{cm}^{-3}}\right)\left(\frac{R_{PDS}}{\text{pc}}\right)^3, \tag{9}$$

we estimate that CRs can produce at most $\sim 4 \times 10^{-12}\, M_\odot$ of ^{10}Be, which is far below the low-mass CCSN yield of $\sim 3.26 \times 10^{-10}\, M_\odot$ (see Table 1 of the main text). Therefore, even

allowing for complications of remnant evolution, CR production inside the remnant would have been a subdominant contribution to the ^{10}Be in the early SS.

Reference [14] considered a remnant interacting with the protosolar cloud and suggested that CR production of ^{10}Be inside the cloud might have provided this SLR to the calcium-aluminum-rich inclusions with Fractionation and Unidentified Nuclear isotope effects (FUN-CAIs). However, it is not clear how this production actually took place when the very small size of the cloud relative to the remnant is taken into account. It is highly desirable to extend the study in Ref. [14] to our proposed low-mass CCSN trigger scenario.

Potential tests for a low-mass CCSN trigger: Li, Be, B. In our proposed scenario, a low-mass CCSN trigger provided the bulk of the ^{10}Be inventory in the early SS as indicated by canonical CAIs. CR production associated with the CCSN remnant might have provided ^{10}Be to FUN-CAIs [14]. Any ^{10}Be production by CRs and solar energetic particles (SEPs) [15, 16] would be in addition to the injection from the CCSN but generally at subdominant levels consistent with the observed variations of ^{10}Be/^{9}Be in canonical CAIs [17–20].

We propose a potential test of the above scenario based on the distinct yield pattern of Li, Be, and B isotopes for the CCSN. Supplementary Table 4 gives the yields of ^{6}Li, ^{7}Li, ^{9}Be, ^{10}B, and ^{11}B for the 11.8 $M_\odot$ model with no fallback (Case 1). The fallback in Cases 2 and 3 causes little change in these results. It can be seen that the CCSN predominantly produces ^{7}Li and ^{11}B, which is a feature of neutrino-induced nucleosynthesis [21]. This is in sharp contrast to the production by CRs or SEPs with much higher energy than CCSN neutrinos. For example, Ref. [14] gave relative number yields of $^{6}\mathrm{Li} : {}^{7}\mathrm{Li} : {}^{9}\mathrm{Be} : {}^{10}\mathrm{B} : {}^{11}\mathrm{B} \sim 1 : 1.8 : 0.11 : 0.43 : 1.1$.

The presence of ^{10}Be in the early SS is established by the correlation between ^{10}B/^{11}B and ^{9}Be/^{11}B, from which the initial values $(^{10}\mathrm{Be}/^{9}\mathrm{Be})_0$ and $(^{10}\mathrm{B}/^{11}\mathrm{B})_0$ at the time of ^{10}Be incorporation are obtained. In our scenario, the low-mass CCSN trigger provided the bulk of the ^{10}Be and $\sim 5\%$ of the ^{11}B in the SS (see Supplementary Table 4). Consequently, we expect that samples with higher $(^{10}\mathrm{Be}/^{9}\mathrm{Be})_0$ would have lower $(^{10}\mathrm{B}/^{11}\mathrm{B})_0$ due to the excess of ^{11}B over ^{10}B that accompanied the ^{10}Be from the CCSN. The variations in $(^{10}\mathrm{B}/^{11}\mathrm{B})_0$ should be at the level of $\sim 5\%$. Such variations are consistent with the data reported in Refs. [19, 20]. It remains to be seen if future meteoritic studies with more samples and better precision can establish the above relationship rigorously, thereby providing a test for the low-mass CCSN trigger.

A correlation between ^{7}Li/^{6}Li and ^{9}Be/^{6}Li was found in a sample with $(^{10}\mathrm{Be}/^9\mathrm{Be})_0 = (8.8 \pm 0.6) \times 10^{-4}$ and interpreted as indicating the presence of the SLR ^{7}Be in the early SS [22]. If true, the extremely short lifetime of 77 days for ^{7}Be would almost certainly require irradiation by SEPs for its production, and by association, the same mechanism may also have produced the ^{10}Be in the sample. However, the above result was disputed and the controversy remains unresolved [23, 24]. Here we propose an alternative explanation for the tantalizing correlation between ^{7}Li/^{6}Li and ^{9}Be/^{6}Li. We note that the low-mass CCSN trigger also provided $\sim 0.8\%$ of the ^{7}Li in the SS but a negligible amount of ^{6}Li. We expect that portions of a sample that received higher amounts of ^{10}Be from the CCSN would also have higher amounts of ^{7}Li. For a uniform $(^{10}\mathrm{Be}/^9\mathrm{Be})_0$ across the sample, the above relationship would translate into an apparent correlation between ^{7}Li/^{6}Li and ^{9}Be/^{6}Li, which nevertheless has nothing to do with the presence of ^{7}Be in the early SS. This explanation can be tested more directly by checking the relationship between ^{7}Li/^{6}Li and $(^{10}\mathrm{Be}/^9\mathrm{Be})_0$ for a wide range of samples. In our scenario, ^{7}Li/^{6}Li should increase with $(^{10}\mathrm{Be}/^9\mathrm{Be})_0$. As only a relatively small amount of ^{7}Li was added by the low-mass CCSN, high-precision measurements are required to check this relationship. Such measurements would provide an additional test for the low-mass CCSN trigger and also help resolve the controversy over the ^{7}Be result.

Supplementary References

[1] Takigawa, A. *et al.* Injection of short-lived radionuclides into the early solar system from a faint supernova with mixing fallback. *Astrophys. J.* **688**, 1382–1387 (2008).

[2] Takahashi, K. & Yokoi, K. Beta-decay rates of highly ionized heavy atoms in stellar interiors. *At. Data Nucl. Data Tables* **36**, 375–409 (1987).

[3] Wasserburg, G. J., Busso, M., Gallino, R. & Nollett, K. M. Short-lived nuclei in the early solar system: Possible AGB sources. *Nucl. Phys. A* **777**, 5–69 (2006).

[4] Wasserburg, G. J., Trippella, O. & Busso, M. Isotope anomalies in the Fe-group elements in meteorites and connections to nucleosynthesis in AGB stars. *Astrophys. J.* **805**, 7 (2015).

[5] Yoshida, T. *et al.* Neutrino-nucleus reaction cross sections for light element synthesis in supernova explosions. *Astrophys. J.* **686**, 448–466 (2008).

[6] Cyburt, R. H. *et al.* The JINA REACLIB database: Its recent updates and impact on type-I X-ray bursts. *Astrophys. J. Suppl. Ser.* **189**, 240–252 (2010).

[7] Shigeyama, T. & Nomoto, K. Theoretical light curve of SN 1987A and mixing of hydrogen and nickel in the ejecta. *Astrophy. J.* **360**, 242–256 (1990).

[8] Clayton, R. N., Grossman, L. & Mayeda, T. K. A component of primitive nuclear composition in carbonaceous meteorites. *Science* **182**, 485–488 (1973).

[9] Boss, A. P. & Keiser, S. A. Who pulled the trigger: A supernova or an asymptotic giant branch star? *Astrophy. J.* **717**, L1–L5 (2010).

[10] Boss, A. P. & Keiser, S. A. Triggering collapse of the presolar dense cloud core and injecting short-lived radioisotopes with a shock wave. III. Rotating three-dimensional cloud cores. *Astrophys. J.* **788**, 20 (2014).

[11] Boss, A. P. & Keiser, S. A. Triggering collapse of the presolar dense cloud core and injecting short-lived radioisotopes with a shock wave. IV. Effects of rotational axis orientation. *Astrophys. J.* **809**, 103 (2015).

[12] Bergin, E. A. & Tafalla, M. Cold dark clouds: The initial conditions for star formation. *Annu. Rev. Astron. Astrophys.* **45**, 339–396 (2007).

[13] Cioffi, D. F., McKee, C. F. & Bertschinger, E. Dynamics of radiative supernova remnants. *Astrophys. J.* **334**, 252–265 (1988).

[14] Tatischeff, V., Duprat, J. & de Séréville, N. Light-element nucleosynthesis in a molecular

cloud interacting with a supernova remnant and the origin of beryllium-10 in the protosolar nebula. *Astrophys. J.* **796**, 124 (2014).

[15] Gounelle, M. *et al.* Extinct radioactivities and protosolar cosmic rays: Self-shielding and light elements. *Astrophys. J.* **548**, 1051–1070 (2001).

[16] Gounelle, M. *et al.* The irradiation origin of beryllium radioisotopes and other short-lived radionuclides. *Astrophys. J.* **640**, 1163–1170 (2006).

[17] McKeegan, K. D., Chaussidon, M. & Robert, F. Incorporation of short-lived ^{10}Be in a calcium-aluminum-rich inclusion from the Allende meteorite. *Science* **289**, 1334–1337 (2000).

[18] MacPherson, G. J., Huss, G. R. & Davis, A. M. Extinct ^{10}Be in Type A calcium-aluminum-rich inclusions from CV chondrites. *Geochim. Cosmochim. Acta* **67**, 3165–3179 (2003).

[19] Wielandt, D. *et al.* Evidence for multiple sources of ^{10}Be in the early solar system. *Astrophys. J.* **748**, L25 (2012).

[20] Srinivasan, G. & Chaussidon, M. Constraints on ^{10}Be and ^{41}Ca distribution in the early solar system from ^{26}Al and ^{10}Be studies of Efremovka CAIs. *Earth Planet. Sci. Lett.* **374**, 11–23 (2013).

[21] Woosley, S. E., Hartmann, D. H., Hoffman, R. D. & Haxton, W. C. The ν-process. *Astrophys. J.* **356**, 272–301 (1990).

[22] Chaussidon, M., Robert, F. & McKeegan, K. D. Li and B isotopic variations in an Allende CAI: Evidence for the in situ decay of short-lived ^{10}Be and for the possible presence of the short-lived nuclide ^{7}Be in the early solar system. *Geochim. Cosmochim. Acta* **70**, 224–245 (2006).

[23] Desch, S. J. & Ouellette, N. Comment on "Li and Be isotopic variations in an Allende CAI: Evidence for the in situ decay of short-lived ^{10}Be and for the possible presence of the short-lived nuclide ^{7}Be in the early solar system," by M. Chaussidon, F. Robert, and K.D. McKeegan. *Geochim. Cosmochim. Acta* **70**, 5426–5432 (2006).

[24] Chaussidon, M., Robert, F. & McKeegan, K. D. Reply to the comment by Desch and Ouellette on "Li and B isotopic variations in an Allende CAI: Evidence for the in situ decay of short-lived ^{10}Be and for the possible presence of the short-lived nuclide ^{7}Be in the early solar system". *Geochim. Cosmochim. Acta* **70**, 5433–5436 (2006).
